# Bisubstrate UDP–peptide conjugates as human O-GlcNAc transferase inhibitors

**DOI:** 10.1042/BJ20131272

**Published:** 2014-01-10

**Authors:** Vladimir S. Borodkin, Marianne Schimpl, Mehmet Gundogdu, Karim Rafie, Helge C. Dorfmueller, David A. Robinson, Daan M. F. vanAalten

**Affiliations:** *MRC Protein Phosphorylation und Ubiquitylation Unit, College of Life Sciences, University of Dundee, Dow Street, Dundee DD1 5EH, U.K.; †Division of Molecular Microbiology, College of Life Sciences, University of Dundee, Dow Street, Dundee DD1 5EH, U.K.; ‡Drug Discovery Unit, College of Life Sciences, University of Dundee, Dow Street, Dundee DD1 5EH, U.K.

**Keywords:** bisubstrate analogue inhibitor, glycosyltransferase, O-GlcNAc, rational drug design, DIPEA, *N*,*N*-di-isopropylethylamine, DMF, dimethylformamide, goblin, OGT bisubstrate-linked inhibitor, h, human, HRMS, high-resolution MS, MP, *p*-methoxyphenyl, OGA, O-GlcNAc hydrolase, OGT, O-GlcNAc:polypeptidyl transferase, TAB1, TGF (transforming growth factor)-β-activated kinase-binding protein 1

## Abstract

Inhibitors of OGT (O-GlcNAc transferase) are valuable tools to study the cell biology of protein O-GlcNAcylation. We report OGT bisubstrate-linked inhibitors (goblins) in which the acceptor serine in the peptide VTPVSTA is covalently linked to UDP, eliminating the GlcNAc pyranoside ring. Goblin1 co-crystallizes with OGT, revealing an ordered C_3_ linker and retained substrate-binding modes, and binds the enzyme with micromolar affinity, inhibiting glycosyltransfer on to protein and peptide substrates.

## Abstract 2

Inhibitors of OGT (O-GlcNAc transferase) are valuable tools to study the cell biology of protein O-GlcNAcylation. We report OGT bisubstrate-linked inhibitors (goblins) in which the acceptor serine in the peptide VTPVSTA is covalently linked to UDP, inhibiting glycosyltransfer on to protein and peptide substrates.

## INTRODUCTION

Reversible post-translational modification of nuclear and cytoplasmic proteins with β-linked O-GlcNAc in metazoa is involved in numerous signal transduction cascades that regulate almost every cellular process [1–3]. O-GlcNAc cycling is governed by a pair of antagonistic enzymes existing as single-copy genes in two to three splice variants, namely OGT (O-GlcNAc:polypeptidyl transferase) and OGA (O-GlcNAc hydrolase). An *ogt* gene knockout in mice has been shown to be lethal at the embryonic level [4]. Aberrant O-GlcNAc profiles on certain proteins are associated with the onset and progression of neurodegenerative disease [5]. Existing inhibitors of OGA have been used to induce cellular hyper-O-GlcNAcylation both *in vitro* and *in vivo* [6]. Conversely, in-depth elucidation of the biological implications of cellular hypo-O-GlcNAcylation is hampered by the dearth of suitable effectors of OGT. To date, only a limited number of OGT inhibitors have been reported, all targeting the UDP-GlcNAc-binding site. The compound BZX {4-methoxyphenyl 6-acetyl-2-oxobenzo[*d*]oxazole-3(2*H*)-carboxylate}, proposed to be a neutral pyrophosphate mimic, was identified as a cell-permeant irreversible inhibitor of hOGT (where h denotes human) [7]. The mechanism of hOGT inhibition with BZX involves cross-linking of the active-site residues Lys^842^ and Cys^917^ with an S-thiocarbamate link [8]. A unique approach to hOGT inhibition was reported previously [9], employing cell-penetrant per-acetylated 4Ac-5S-GlcNAc (2-acetamido-2-deoxy-5-thio-D-glucopyranose). Once inside the cell, this compound is deacetylated by non-specific esterases producing the free thiosugar 5S-GlcNAc, which is then a substrate for the UDP-GlcNAc biosynthetic pathway, leading to incorporation of the thiosugar into the OGT donor substrate analogue UDP-5S-GlcNAc. The latter inhibits hOGT *in vitro* (*K*_i_=8 μM) and *in vivo*, although the basis for selectivity over other GlcNAc transferases remains to be fully explained [9]. A set of non-hydrolysable substrate analogues including the glycosyl thiophosphate (UDP-S-GlcNAc) and C-glycosylphosphonate (UDP-C-GlcNAc) have also been reported [10]. Although these compounds moderately inhibited hOGT, they are not expected to be selective probes. In common with many other glycosyltransferases, OGT is subject to product inhibition, and, accordingly, the most potent inhibitor of hOGT reported to date is the reaction product UDP (*K*_d_ 0.5 μM) [11]. However, this product inhibition is difficult to exploit for inhibitor design. Not only would the charged nature of the compound render it cell-impermeant, but, in addition, UDP is a central metabolite involved in nucleic acid (RNA) biosynthesis, as well as in the activation of monosaccharides as glycosyl donors for glycoconjugate biosynthesis (six out of the nine mammalian nucleotide sugars are UDP-sugars). UDP analogues consequently have the potential to interact with diverse classes of enzymes such as oxidoreductases, transferases, hydrolases, lyases and isomerases. Bisubstrate inhibitors would offer a means of engineering selectivity by capitalizing on the specific combination of substrates.

Recent structural snapshots of ternary hOGT complexes with substrate/product analogues have uncovered invaluable insights into the hOGT catalytic mechanism [11,12]. Upon binding of the acceptor substrate, hitherto solvent-exposed parts of the bound UDP-GlcNAc engage directly with the peptide backbone, aligning the incoming nucleophile and the anomeric carbon in a glycosyltransfer-competent conformation. This conformation also brings together the acceptor serine and the pyrophosphate moiety, an interaction that has been proposed to be essential for catalysis [11]. In the present study, we exploit these data for the rational design of new OGT bisubstrate inhibitors that combine elements of both substrates; an approach expected to result in selective inhibition of the enzyme compared with inhibitors limited to targeting the donor-binding site alone.

## MATERIALS AND METHODS

### Chemical synthesis

**Scheme 1:**
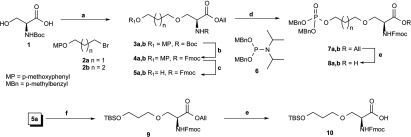
Synthesis of the ‘stretched serine’ building blocks (a) (i) NaH, Bu4NI (tetra-n-butylammonium iodide), **2a** or **2b**, DMF, 0°C then room temperature, 16 h, (ii) AllBr (allyl bromide), DIPEA, DMF, room temperature, 16 h, 30% for two steps; (b) (i) 95% TFA (trifluoroacetic acid), water, DCM (dichloromethane), room temperature, 1 h, (ii) FmocCl (Fmoc chloride), DIPEA, DCM, room temperature, 16 h, 90% for two steps; (c) CAN [ammonium cerium(IV) nitrate], acetonitrile, THF (tetrahydrofuran), water, room temperature, 1 h, 92%; (d) **6**, 4,5-dicyanoimidazole, acetonitrile, room temperature, 1 h then mCPBA (m-chloroperoxybenzoic acid), 0°C, 1 h, 85%; (e) Pd[(PPh_3_)]_4_ [tetrakis(triphenylphosphine)palladium(0)], morpholine, THF then Dowex 50WX8-100 H^+^, 100%; (f) TBSCl (t-butyl dimethylchlorosilane), DIPEA, DMAP (4-dimethylaminopyridine), DCM, room temperature,16 h, 92%. Boc, t-butoxycarbonyl.

**Scheme 2:**
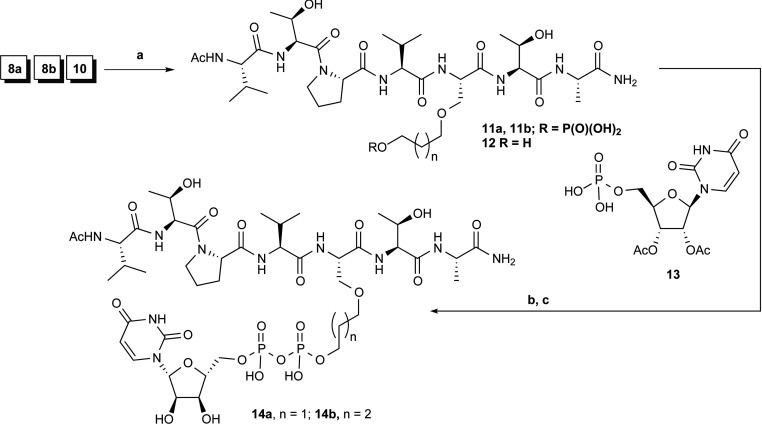
Synthesis of UDP–peptide conjugates and ‘linker-only’ peptide (a) Microwave-assisted Fmoc SPPS (solid-phase peptide synthesis); (b) (i) **13**, CDI (1,1′-carbonylbisimidazole), DMF (dimethylformamide), room temperature, 20 h then methanol then **8a/8b**, room temperature 16 h; (c) methanol, triethylamine, water, room temperature, 16 h then HPLC.

**Scheme 3:**
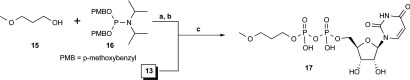
Synthesis of the ‘linker-only’ UDP derivative (a) **16**, 4,5-dicyanoimidazole, acetonitrile, room temperature, 1 h, then (b) I_2_/Py (pyridine), 0°C, 1 h then **13**, room temperature, 16 h; (c) methanol, triethylamine, water, room temperature, 16 h, then size-exclusion chromatography 40%.

Inhibitors were synthesized as outlined in schemes 1–3; for a full description, see the Supplementary Online Data at http://www.biochemj.org/bj/457/bj4570497add.htm. Characterization by NMR and MS, **14a**: ^31^P NMR (202 MHz, ^2^H_2_O) δ −11.08 (d, *J*_Pα,Pβ_ 26.6 Hz), −11.56 (d, *J*_Pα,Pβ_ 26.6 Hz); HRMS (high-resolution MS) (*m*/*z*) [*M*−H]^−^=1157.4171. **14b**: ^31^P NMR (202 MHz, ^2^H_2_O) δ −10.97 (d, *J*_Pα,Pβ_ 27.2 Hz), −11.53 (d, *J*_Pα,Pβ_ 27.2 Hz); HRMS (*m*/*z*) [*M*−H]^−^=1171.4343.

### Protein crystallography

Hanging-drop crystallization experiments with drops containing 2 μl of reservoir solution [1.45 M K_2_HPO_4_, 10 mM EDTA and 1% (w/v) xylitol] and 2 μl of 100 μM hOGT-(312–1031) (purified as described previously [11]) and 1 mM goblin1 (**14a**) in 20 mM Tris/HCl (pH 8.5) and 0.5 mM THP [tris-(3-hydroxypropyl)phosphine] gave hexagonal rod-shaped crystals within 3–4 days at 22°C. Crystals were cryoprotected by 2 s of immersion in a saturated Li_2_SO_4_ solution before flash-freezing in liquid nitrogen. Diffraction data were collected at Diamond Light Source beamline I03. Data were processed with Xia2 and scaled to 3.15 Å (1 Å=0.1 nm) using SCALA [13]. The structure was solved by molecular replacement with PDB code 4AY5 and refined with REFMAC [14]. Model building was performed with Coot [15]. Ligand topology was provided by PRODRG [16].

### *In vitro* glycosylation of hTAB1

Reaction mixtures containing 1 μM TAB1-(7–402) protein [TAB1 is TGF (transforming growth factor)-β-activated kinase-binding protein 1] (purified as described previously [17]), 0.125 μM hOGT-(312–1031) and 10 μM UDP-GlcNAc in a buffer of 50 mM Tris/HCl (pH 7.5) and 1 mM DTT were incubated at 37°C for 90 min, separated by SDS/PAGE (10% gels) and transferred on to nitrocellulose membranes. Membranes were probed with a TAB1-gSer^395^ (O-GlcNAcylated Ser^395^) O-GlcNAc site-specific antibody [18], followed by an IR800-labelled secondary antibody and analysed using a LI-COR Odyssey scanner and associated quantification software. Data were fitted to a four-parameter equation for dose-dependent inhibition using GraphPad Prism 5.0.

### Steady-state kinetics

Reactions contained 50 nM hOGT-(312–1031) in 50 mM Tris/HCl (pH 7.5), 0.1 mg/ml BSA, 10 μM sodium dithionate and 10 μM peptide (KKENSPAVTPVSTA) and various amounts of inhibitors in a total volume of 100 μl. Reaction mixtures were pre-incubated for 15 min and started by addition of UDP-GlcNAc to a final concentration of 3.2 μM. After 30 min of incubation at 22°C, assays were stopped by adding 200 μl of 25 mM Hepes (pH 7.4), 10 mM NaCl, 50% (v/v) methanol and 15 μM fluorophore, a UDP-sensitive xanthene-based Zn(II) complex prepared as described in [19,20]. Product formation was detected fluorimetrically on a Gemini EM fluorescence microplate reader (Molecular Devices) at excitation and emission wavelengths of 485 nm and 530 nm respectively. A non-linear regression curve fit was performed with Prism.

### Biolayer interferometry

Measurements were made on a ForteBio Octet RED384 instrument at 25°C. Biotinylated hOGT-(312–1031) was prepared at 25 μg/ml in TBS (25 mM Tris/HCl, pH 7.5, and 150 mM NaCl) buffer containing 1 mM DTT and immobilized on superstreptavidin biosensors. Free streptavidin sites were blocked by incubation with biocytin. A parallel set of superstreptavidin biosensors were prepared with biotinylated streptavidin to act as a control. The assay was carried out in 384-well plates with a sample volume of 100 μl. Inhibitor solutions were prepared from solid stocks and dissolved in assay buffer and a concentration series of 3-fold dilutions from a top concentration of 100 μM was created. Cycles for analysis involved obtaining a 30 s baseline followed by a 60 s association step and a 120 s dissociation step. The assay was repeated with the reference biosensors to correct for non-specific interactions and the entire assay was repeated in triplicate for both compounds. Data were processed and kinetic parameters were calculated using ForteBio software.

## RESULTS AND DISCUSSION

**Figure 1:**
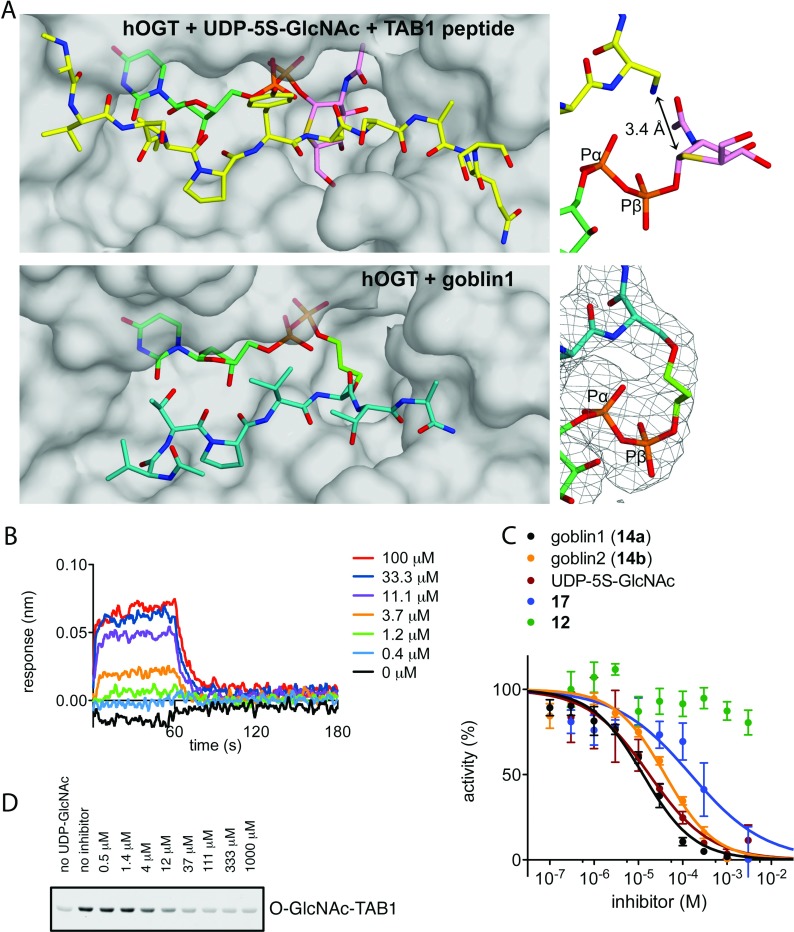
Analysis of goblin1 binding and inhibition (**A**) Structure of hOGT Michaelis complex [11] (PDB code 4AY6) and the hOGT–goblin1 complex. hOGT is shown as a molecular surface. The donor substrate UDP-5S-GlcNAc (pink/turquoise carbon atoms), the acceptor peptide (yellow carbon atoms) and goblin1 (green carbon atoms) are shown as stick models. On the right, a close-up view of the compounds is shown, centred on the linker incorporated in goblin1. For the hOGT–goblin1 complex, an unbiased (i.e. before incorporating of any ligand) four-fold NCS (non-crystallographic symmetry) averaged *F*_o_−*F*_c_ electron density map is shown, contoured at 3.5σ. Full goblin1 *F*_o_−*F*_c_ maps are shown in Supplementary Figure S3 at http://www.biochemj.org/bj/457/bj4570497add.htm. (**B**) Biolayer interferometry was used to measure binding affinity of hOGT for goblin1 (*K*_d_ 7.9 μM). A representative binding profile is shown, obtained from a concentration series of goblin1 interacting with hOGT. Further data and curve fits are shown in Supplementary Figure S1 at http://www.biochemj.org/bj/457/bj4570497add.htm. (**C**) IC_50_ determination of goblin1 and goblin2 in comparison with the inhibitor UDP-5S-GlcNAc and fragments of the bisubstrate inhibitor, compounds **12** and **17**. Data points are means±S.E.M. for three experiments. (**D**) Goblin1 inhibition of glycosylation of substrate protein TAB1, as detected by anti-O-GlcNAc Western blot. Densitometric analysis and IC_50_ determination are shown in Supplementary Figure S2 at http://www.biochemj.org/bj/457/bj4570497add.htm.

Inspection of the ternary hOGT–UDP-5S-GlcNAc–TAB1 peptide complex [11] shows that the anomeric carbon of the sugar is positioned at a distance of 3.4 Å from the modified peptide side chain (Figure 1A). Biophysical determination of the binding affinity for product and substrate revealed a 30-fold higher *K*_d_ for UDP-GlcNAc than for UDP [11], suggesting that, in the absence of an acceptor, the sugar moiety does not positively contribute to the binding affinity.

We consequently envisaged a set of bisubstrate OGT inhibitors in the form of UDP–peptide conjugates in which UDP is coupled to a variable peptide subunit by a suitable linker. The weakly binding GlcNAc moiety was omitted from the inhibitor structure and replaced with a linear three- or four-carbon tether to retain the spatial arrangement of UDP and peptide as observed in the Michaelis complex. The resulting compounds were named goblin1 (OGT bisubstrate-linked inhibitor 1) and goblin2 respectively.

Synthesis of the target compounds entailed the preparation of phosphorylated ‘stretched serine’ derivatives (Scheme 1) suitable for Fmoc solid-phase peptide synthesis and peptide assembly followed by reaction of the phosphorylated peptides with activated nucleoside monophosphate (Scheme 2). Synthesis of analogous peptide–nucleotide polyphosphate conjugates as protein kinase bisubstrate inhibitors using an on-resin phosphorylation–pyrophosphorylation protocol has been reported previously [21]. In the first instance, we opted for off-resin pyrophosphorylation for the reason of operational and analytical convenience. We established a robust, albeit moderately yielding, two-step procedure for the preparation of the key ‘stretched serine’ allyl esters **3a/b** by alkylation [22] of the dianion generated from the commercially available N-Boc (*N*-t-butoxycarbonyl) serine **1** with MP (*p*-methoxyphenyl)-protected 3-bromopropan-1-ol or 4-bromobutan-1-ol **2a/2b** [NaH, DMF (dimethylformamide)] followed by esterification with allyl bromide/DIPEA (*N*,*N*-di-isopropylethylamine) (Scheme 1). Attempted alkylation of **1** under phase-transfer conditions [23] was unsuccessful, whereas more elaborate synthetic schemes proved to be inefficient. Ensuing N-protective group remodelling as well as removal of the terminal hydroxy group protection (**3a/3b**→**4a/4b**→**5a/5b**) was ensured by the perfect orthogonality of the MP protecting group, resulting in nearly quantitative yield for three steps. Finally, installation of the phosphate group with acid-labile MBn (*p*-methylbenzyl) protection and deblocking of the C-terminus yielded the required phosphorylated ‘stretched serine’ building blocks **8a/8b** with very good overall efficiency. Careful choice of the phosphate protecting group was essential, as the traditional dibenzyl phosphate protection failed to be cleanly removed under the peptide cleavage conditions. Full details of the synthesis are given in the Supplementary Online Data.

Peptide synthesis was performed on a microwave-assisted CEM Liberty instrument using RinkAmide MBHA (4-methy-lbenzhydrylamine) resin and HCTU [*O*-(1*H*-6-chlorobenzo-triazole-1-yl)-1,1,3,3-tetramethyluronium hexafluorophosphate] as the coupling agent (for details, see the Supplementary Online Data). The synthesis of the UDP–peptide conjugates **14a/b** is illustrated in Scheme 2. Crude phosphopeptides **11a/b** were reacted with a 10-fold excess of 2′,3′-*O*-diacetyl-UMP imidazolide prepared from triethylammonium salt **13** and CD1 (1,1′-carbonylbisimidazole) overnight, deacetylated with methanol/triethylamine/water and finally purified by reverse-phase HPLC to furnish the targeted UDP–peptide conjugates **14a** (goblin1) and **14b** (goblin2) in fair yield. The identity of the newly synthesized compounds was confirmed by TOF–ESI–MS and NMR spectroscopy.

To investigate the contribution of the separate parts of the UDP–peptide conjugate towards inhibition potency, we synthesized ‘linker-only’ peptide **12** using ‘stretched serine’ building block **10** (Scheme 1). We also prepared the matching ‘linker-only’ UDP analogue **17** starting from 3-methoxypropanol taking advantage of the one-pot/three-step pyrophosphorylation procedure mediated by di-(*p*-methoxybenzyl)-*N*,*N*-di-isopropylphosphoramidite **16** [24] (Scheme 3).

The affinity of goblin1 and goblin2 for hOGT was evaluated by biolayer interferometry (Figure 1B, and Supplementary Figure S1 at http://www.biochemj.org/bj/457/bj4570497add.htm) yielding *K*_d_ values of 7.9 and 4.9 μM respectively. *In vitro* glycosylation of a peptide substrate was inhibited in a dose-dependent manner with an IC_50_ value of 18 μM for goblin1 and 40 μM for goblin2 (Figure 1C). Furthermore, the ability of goblin1 to inhibit O-GlcNAcylation of a well-characterized human substrate protein, TAB1 [18], was investigated by Western blotting employing a TAB1 O-GlcNAc Ser^395^ site-specific antibody (Figure 1D, and Supplementary Figure S2 at http://www.biochemj.org/bj/457/bj4570497add.htm). Dose-dependent inhibition of hOGT activity was observed, and densitometric quantification allowed the calculation of an IC_50_ value of 8 μM (Supplementary Figure S2). In a set of control experiments with ‘linker-only’ compounds **12** and **17**, we observed only weak hOGT inhibition by **17** (IC_50_ 300 μM) whereas ‘linker-only’ peptide **12** was proved to be neither a substrate nor an inhibitor (Figure 1C). Attempts to inhibit OGT in cells appeared unsuccessful, probably due to the size/negative charge of the compounds.

To confirm the binding mode of goblin1, we co-crystallized the compound with hOGT and determined the crystal structure of the complex (Supplementary Table S1 at http://www.biochemj.org/bj/457/bj4570497add.htm). Electron density difference maps at 3.15 Å, improved by 4-fold non-crystallographic averaging (Supplementary Figure S3 at http://www.biochemj.org/bj/457/bj4570497add.htm), revealed unambiguous density for the entire compound, including the ordered C_3_ linker (Figure 1A). As envisaged, UDP adopts the same conformation as observed in the hOGT Michaelis complex (Figure 1A; maximum atom shift, 0.6 Å) and the peptide occupies the −4 to +2 subsites with a similar backbone conformation (maximum backbone atom shift near O-GlcNAc site, 0.9 Å). The three-carbon linker connects the two components apparently without introducing any strain, allowing both the UDP moiety and the peptide part of the inhibitor to adopt the optimal position in the binding site, effectively mimicking the natural substrates.

One of the main objectives of combining components of donor and acceptor substrate into a bisubstrate inhibitor is the expected improved selectivity of such a construct when compared with inhibitors that compete with a single substrate only. Although accessing the selectivity of the novel OGT bisubstrate inhibitors over an exhaustive panel of GlcNAc transferases is beyond the scope of the present study, we were able to establish that goblin1 is not an inhibitor of the GlcNAc transferase *Sm*NodC (*Sinorhizobium meliloti* NodC) (Supplementary Figure S4 at http://www.biochemj.org/bj/457/bj4570497add.htm).

### Concluding remarks

O-GlcNAc modification of proteins is abundant and essential, yet its precise cellular function remains to be uncovered. The development of small-molecule inhibitors of OGT is a prerequisite for the elucidation of the biological role of O-GlcNAc, and eventually the therapeutic intervention in diseases involving dysregulation of O-GlcNAc. With multisubstrate enzymes, such as OGT, specific inhibition can rarely be achieved with ligands that compete solely with one of the substrates. Peptide–nucleotide polyphosphate conjugates have been reported as bisubstrate inhibitors of protein kinases in the early 1990s; the discovery recently led to the identification of low-nanomolar cell-penetrant inhibitors with neutral linkers replacing the polyphosphate chain [25]. The pioneering work of Palcic et al. [26] introduced the concept of bisubstrate inhibition for glycosyltransferases, targeting fucosyltransferases [27].

In the present paper, we have reported the first examples of functional OGT bisubstrate inhibitors featuring a short linker replacing the GlcNAc moiety of the donor substrate and covalently connecting a uridyl diphosphate ‘binding anchor’ to an acceptor peptide. The resulting compounds, goblin1 and goblin2, exhibit low-micromolar affinity for OGT and are capable of inhibiting glycosylation of peptide as well as protein substrates *in vitro*. The crystal structure of the hOGT–goblin1 complex reveals the structural mimicry offered by the inhibitor, with both UDP and peptide moieties retaining the alignment observed in the Michaelis complex. As expected for a bisubstrate inhibitor, both fragments add synergistically to the inhibition: the ‘linker-only’ derivative of UDP was shown to be an order of magnitude less potent an inhibitor, whereas the ‘linker-only’ acceptor peptide was neither a substrate for the OGT nor an inhibitor. The goblin scaffold allows for further increases in potency by optimization of linker length/type, peptide sequence and nucleotide modifications, whereas cell penetrance could be addressed by extending the peptide backbone with cell-penetrating peptide/peptoid motifs [28,29] potentially bearing intracellular localization signals, to provide tools for studying OGT function *in vivo*.

## Online data

Supplementary data
