# Supplementary material for: Bisubstrate UDP–peptide conjugates as human O-GlcNAc transferase inhibitors
Source: Biochem J. 2014 Jan 10;457(Pt 3):497–502. doi: 10.1042/BJ20131272 (PMC3927924; doi:10.1042/BJ20131272)
Supplement: Supplementary data [file bj4570497add.pdf]

BJ ChemBio

Biochem. J. (2014) **457**, 497–502 (Printed in Great Britain) doi:10.1042/BJ20131272

online data

SUPPLEMENTARY ONLINE DATA

Bisubstrate UDP–peptide conjugates as human O-GlcNAc transferase inhibitors

Vladimir S. BORODKIN*, Marianne SCHIMPL*, Mehmet GUNDOGDU*, Karim RAFIE*, Helge C. DORFMUELLER*†, David A. ROBINSON‡ and Daan M. F. VAN AALTEN*†[1]

*MRC Protein Phosphorylation und Ubiquitylation Unit, College of Life Sciences, University of Dundee, Dow Street, Dundee DD1 5EH, U.K.
†Division of Molecular Microbiology, College of Life Sciences, University of Dundee, Dow Street, Dundee DD1 5EH, U.K.
‡Drug Discovery Unit, College of Life Sciences, University of Dundee, Dow Street, Dundee DD1 5EH, U.K.

SYNTHESIS METHODS

Compound 3a

To a solution of the SerBocOH (*N*-t-butoxycarbonylserine) (2.25 g, 11 mmol) in DMF (40 ml) 60 % sodium hydride suspension in oil (0.88 g, 22 mmol) was added sequentially in two equal portions at 0 °C (ice-bath). When gas evolution ceased, the reaction was removed from the cooling bath and stirred at room temperature until gas evolution ceased (1 min). The reaction was returned to the ice-bath and tetra-n-butylammonium iodide (0.369 g, 1 mmol) and a solution of 1-(3-bromopropoxy)-4-methoxybenzene **2** (3.23 g, 13.2 mmol) in DMF (15 ml) were added in a succession. The reaction was removed from the ice-bath and stirred at room temperature for 16 h. The reaction was quenched by careful addition of a few drops of 10 % citric acid solution, diluted with ethyl acetate and washed successively with 10 % citric acid and water. The aqueous layers were back extracted with the ethyl acetate two times more. The combined organic layer was dried and concentrated.

The residue was dissolved in DMF (25 ml) and treated with allyl bromide (1.3 ml, 15 mmol) in the presence of DIPEA (2.6 ml, 15 mmol) at room temperature for 16 h. The reaction was diluted with methanol and concentrated. The residue was partitioned between ethyl acetate and 1 M HCl, and the layers were separated. The organic layer was successively washed with water and a mixture of saturated $NaHCO_3$ solution and brine. The aqueous layers were back-extracted with ethyl acetate. The combined organic layer was dried and concentrated. The residue was adsorbed on to silica and purified by flash chromatography in petroleum ether/ethyl acetate 5–10–20 % to give 1.37 g (3.36 mmol, 30 %) of the target product as clear oil.

$[\alpha]_D = +4.7°$ (c 1.0 in chloroform), ^{1}H NMR (500 MHz, [^{2}H]chloroform) δ 6.82 (s, 4H), 5.86 (ddt, J 17.2, 10.5, 5.7 Hz, 1H), 5.42 (d, J 8.9 Hz, 1H), 5.29 (dq, J 17.3, 1.6 Hz, 1H), 5.19 (dq, J 10.4, 1.3 Hz, 1H), 4.66–4.55 (m, 2H), 4.45 (dt, J 8.8, 3.2 Hz, 1H), 3.95 (td, J 6.2, 1.0 Hz, 2H), 3.91–3.87 (m, 1H), 3.76 (s, 3H), 3.68 (dd, J 9.5, 3.3 Hz, 1H), 3.61 (qt, J 9.5, 6.1 Hz, 2H), 1.98 (p, J 6.2 Hz, 2H), 1.45 (s, 9H); ^{13}C NMR (126 MHz, [^{2}H]chloroform) δ 170.4, 155.5, 153.8, 153, 131.67, 118.4, 115.4, 114.6, 79.9, 70.8, 68, 65.9, 65.1, 55.7, 54.1, 29.5, 28.3. HRMS (m/z), $[M+H]^+$ calculated for $C_{21}H_{32}NO_7$, 410.2179; found 410.2183.

Compound 3b

$[\alpha]_D = +2.2°$ (c 1.0 in chloroform); ^{1}H NMR (500 MHz, [^{2}H]chloroform) δ 6.84 (s, 4H), 5.99–5.85 (m, 1H), 5.38 (d, 1H), 5.37–5.32 (m, 1H), 5.27–5.23 (m, 1H), 4.75–4.59 (m, 2H), 4.47 (dt, J 8.6, 3.4 Hz, 1H), 3.92 (t, J 6.2 Hz, 2H), 3.88 (dd, J 9.4, 3.3 Hz, 1H), 3.79 (s, 3H), 3.68 (dd, J 9.4, 3.4 Hz, 1H), 3.57–3.45 (m, 2H), 1.85–1.76 (m, 2H), 1.77–1.69 (m, 2H), 1.48 (s, 9H); ^{13}C NMR (126 MHz, [^{2}H]chloroform) δ 170.5, 155.5, 153.8, 153.2, 131.7, 118.4, 115.4, 114.5, 80, 71.1, 70.7, 68.4, 65.9, 55.7, 54.2, 28.3, 26.1. HRMS (m/z), $[M+H]^+$ calculated for $C_{22}H_{35}NO_7$, 425.2414; found 425.2422.

Compound 4a

To a cold (ice-bath) stirred solution of **3a** (1.18 g, 2.9 mmol) in DCM (dichloromethane) (15 ml) was added 95 % aqueous TFA (trifluoroacetic acid) (1.5 ml). The reaction was kept for 2 h at room temperature, diluted with toluene (10 ml) and concentrated. The residue was dissolved in a mixture of chloroform and toluene (1:3, v/v) (10 ml) and concentrated. This procedure was repeated once more. The residue was dissolved in DCM (20 ml) and treated with an excess of DIPEA (2 ml) and FmocCl (Fmoc chloride) (0.905 g, 3.5 mmol) at 0 °C (ice-bath). The reaction was kept at room temperature for 16 h, quenched with methanol (0.1 ml), stirred for 30 min and concentrated. The residue was dissolved in DCM and successively washed with 1 M HCl, water and a mixture of $NaHCO_3$ solution and brine. Aqueous layers were back-extracted with DCM. The combined organic layer was dried and concentrated. The residue was adsorbed on to silica gel and purified by flash chromatography in petroleum ether/ethyl acetate 5–10–20 % to give 1.42 g (2.67 mmol, 92 %) of the target product as crystalline solid. The analytical sample was crystallized from toluene/petroleum ether.

Melting point 103 °C; $[\alpha]_D = +11.4°$ (c 1.00 in chloroform); ^{1}H NMR (500 MHz, [^{2}H]chloroform) δ 7.67 (d, J 7.5 Hz, 2H), 7.52 (t, J 6.6 Hz, 2H), 7.31 (t, J 7.5 Hz, 2H), 7.22 (td, J 7.4, 1.2 Hz, 2H), 6.78–6.68 (m, 4H), 5.78 (ddt, J 16.2, 10.9, 5.7 Hz, 1H), 5.62 (d, J 8.7 Hz, 1H), 5.21 (dq, J 17.1, 1.6 Hz, 1H), 5.11 (dq, J 10.4, 1.3 Hz, 1H), 4.54 (dt, J 5.7, 1.5 Hz, 2H), 4.49–4.42 (m, 2H), 4.34 (dd, J 10.6, 7.2 Hz, 1H), 4.26 (dd, J 10.6, 7.3 Hz, 1H), 4.15 (t, J 7.3 Hz, 1H), 3.87 (td, J 6.2, 1.8 Hz, 2H), 3.84 (dd, J 9.5, 3.2 Hz, 1H), 3.64 (s, 3H), 3.55 (qt, J 9.6, 6.2 Hz, 2H), 1.91 (p, J 6.1 Hz, 2H); ^{13}C NMR (126 MHz, [^{2}H]chloroform) δ 170.1, 156, 153.8, 153, 143.9, 143.8, 141.3, 131.6, 127.7, 127.1, 125.22, 120, 118.6, 115.4, 114.7, 76.9, 70.7, 68.2, 67.2, 65.1, 55.7, 54.6, 47.2, 29.5. HRMS (m/z), $[M+H]^+$ calculated for $C_{31}H_{34}NO_7$, 532.2335; found 532.2331.

[1] To whom correspondence should be addressed (email dmfvanaalten@dundee.ac.uk).
Co-ordinates and structure factors for human O-GlcNAc transferase complexed to the Goblin1 inhibitor have been deposited in the PDB under code 4CDR.

© 2014 The Author(s)
The author(s) has paid for this article to be freely available under the terms of the Creative Commons Attribution Licence (CC-BY) (http://creativecommons.org/licenses/by/3.0/) which permits unrestricted use, distribution and reproduction in any medium, provided the original work is properly cited.

Biochemical Journal www.biochemj.org

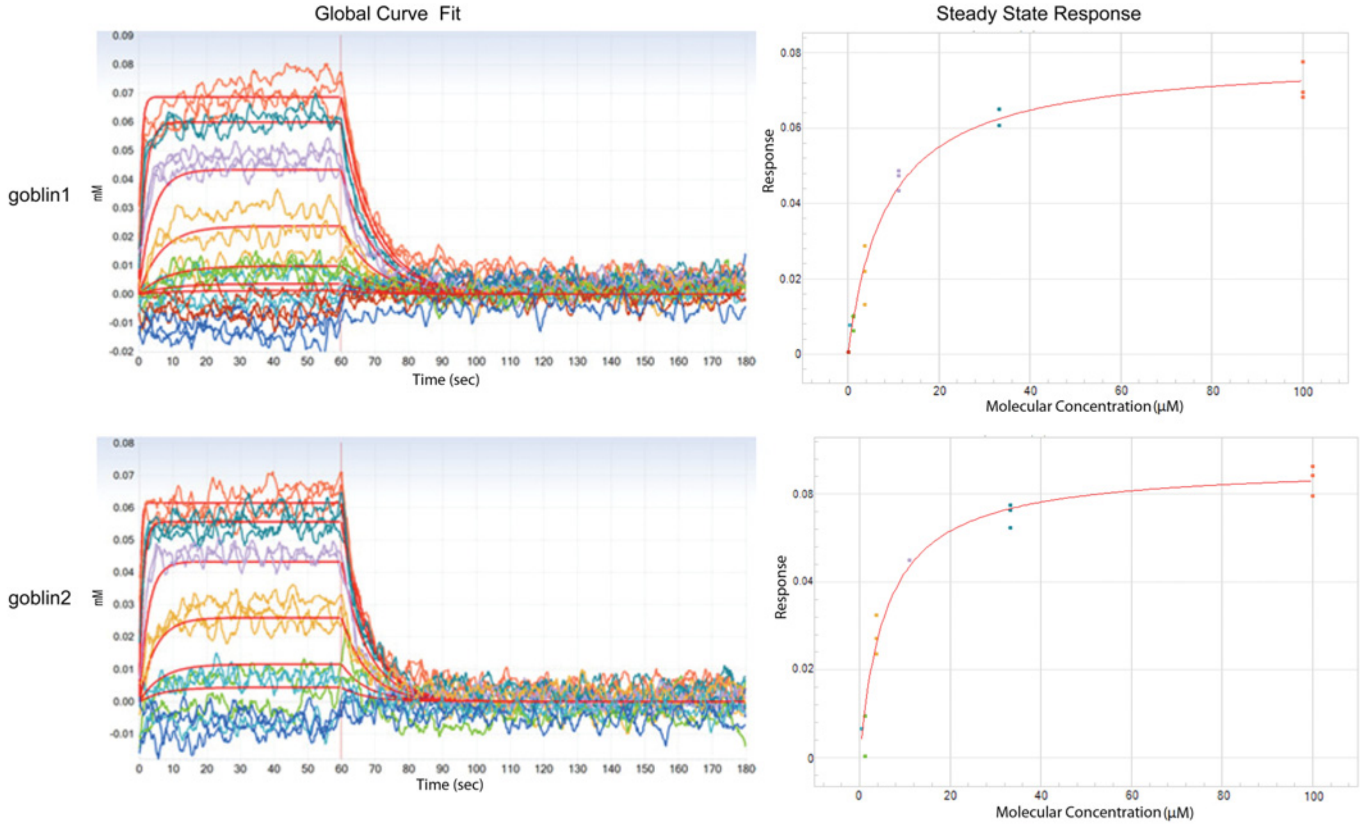

Compound	K_d	k_{on}	k_{off}	Steady State K_d
goblin1	7.9×10^{-6} M	1.3×10^{4} M/s^{-1}	1.0×10^{-1} s^{-1}	8.6×10^{-6} M
goblin2	4.9×10^{-6} M	2.6×10^{4} M/s^{-1}	1.3×10^{-1} s^{-1}	5.0×10^{-6} M

Figure S1 Biolayer interferometry sensograms

Binding profiles obtained and kinetic parameters calculated for interactions between hOGT and goblin1 and goblin2. Concentration series of each compound (3-fold serial dilution, top concentration 100 μM) were prepared in triplicate. Left-hand panels: binding profiles and global curve fitting from which the kinetic parameters were calculated. Right-hand panels: steady-state binding response from which the steady-state K_d was calculated.

Compound 4b

$[\alpha]_D = +13.5°$ (c 1.00 in chloroform); ^{1}H NMR (500 MHz, [^{2}H]chloroform) δ 7.80 (dd, J 7.4, 1.1 Hz, 2H), 7.70–7.60 (m, 2H), 7.43 (tt, J 7.4, 0.9 Hz, 2H), 7.34 (td, J 7.5, 1.2 Hz, 2H), 6.85 (s, 4H), 5.94 (ddt, J 16.4, 10.9, 5.7 Hz, 1H), 5.70 (d, J 8.7 Hz, 1H), 5.37 (dq, J 17.2, 1.7 Hz, 1H), 5.32–5.22 (m, 1H), 4.79–4.65 (m, 2H), 4.58 (dt, J 8.7, 3.3 Hz, 1H), 4.46 (dd, J 10.6, 7.2 Hz, 1H), 4.40 (dd, J 10.6, 7.3 Hz, 1H), 4.28 (t, J 7.3 Hz, 1H), 3.94 (t, J 6.0 Hz, 3H), 3.79 (s, 3H), 3.62–3.48 (m, 2H), 1.80 (dq, J 25.9, 7.3 Hz, 4H). HRMS (m/z), $[M+H]^+$ calculated for $C_{32}H_{37}NO_7$, 547.2570; found 547.2558.

Compound 5a

To a stirred solution of **4a** (1.39 g, 2.61 mmol) in a mixture of THF (tetrahydrofuran)/acetonitrile (1:1, v/v) (15 ml) a solution of ammonium cerium(IV) nitrate (3 g, 5.47 mmol) in water (7.5 ml) was added at room temperature. The reaction was stirred for 20 min, diluted with 10 % EDTA solution and ethyl acetate and the layers were separated. The organic layer was washed successively with 10 % aqueous $Na_2S_2O_5$ solution, water and a mixture of saturated $NaHCO_3$ solution and brine. The aqueous layers were back-extracted with ethyl acetate. The combined organic layer was dried and concentrated. The residue was adsorbed on to silica gel and purified by flash chromatography in (petroleum ether/DCM, 4:1, v/v)/ethyl acetate 5→45 % to give 1.1 g (2.58 mmol, ∼100 %) of the target product as tan-coloured crystals. The analytical sample was crystallized from chloroform/petroleum ether.

Melting point 73 °C; $[\alpha]_D = +11.3°$ (c 1.00 in chloroform); ^{1}H NMR (500 MHz, [^{2}H]chloroform) δ 7.76–7.62 (m, 2H), 7.54 (t, J 6.9 Hz, 2H), 7.32 (tt, J 7.6, 1.4 Hz, 3H), 7.24 (tt, J 7.3, 1.3 Hz, 2H), 5.84 (ddt, J 16.4, 10.4, 5.7 Hz, 1H), 5.72 (d, J 8.6 Hz, 1H), 5.26 (dq, J 17.2, 1.5 Hz, 1H), 5.18 (dt, J 10.4, 1.3 Hz, 1H), 4.65–4.55 (m, 2H), 4.48 (dt, J 8.6, 3.4 Hz, 1H), 4.35 (dd, J 10.6, 7.2 Hz, 1H), 4.29 (dd, J 10.6, 7.2 Hz, 1H), 4.16 (t, J 7.2 Hz, 1H), 3.82 (dd, J 9.7, 3.5 Hz, 1H), 3.66–3.61 (m, 3H), 3.57 (ddd, J 9.4, 6.5, 5.1 Hz, 1H), 3.51 (ddd, J 9.4, 6.7, 5.1 Hz, 1H), 1.80–1.62 (m, 2H); ^{13}C NMR (126 MHz, [^{2}H]chloroform) δ 170.1,

© 2014 The Author(s)
The author(s) has paid for this article to be freely available under the terms of the Creative Commons Attribution Licence (CC-BY) (http://creativecommons.org/licenses/by/3.0/) which permits unrestricted use, distribution and reproduction in any medium, provided the original work is properly cited.

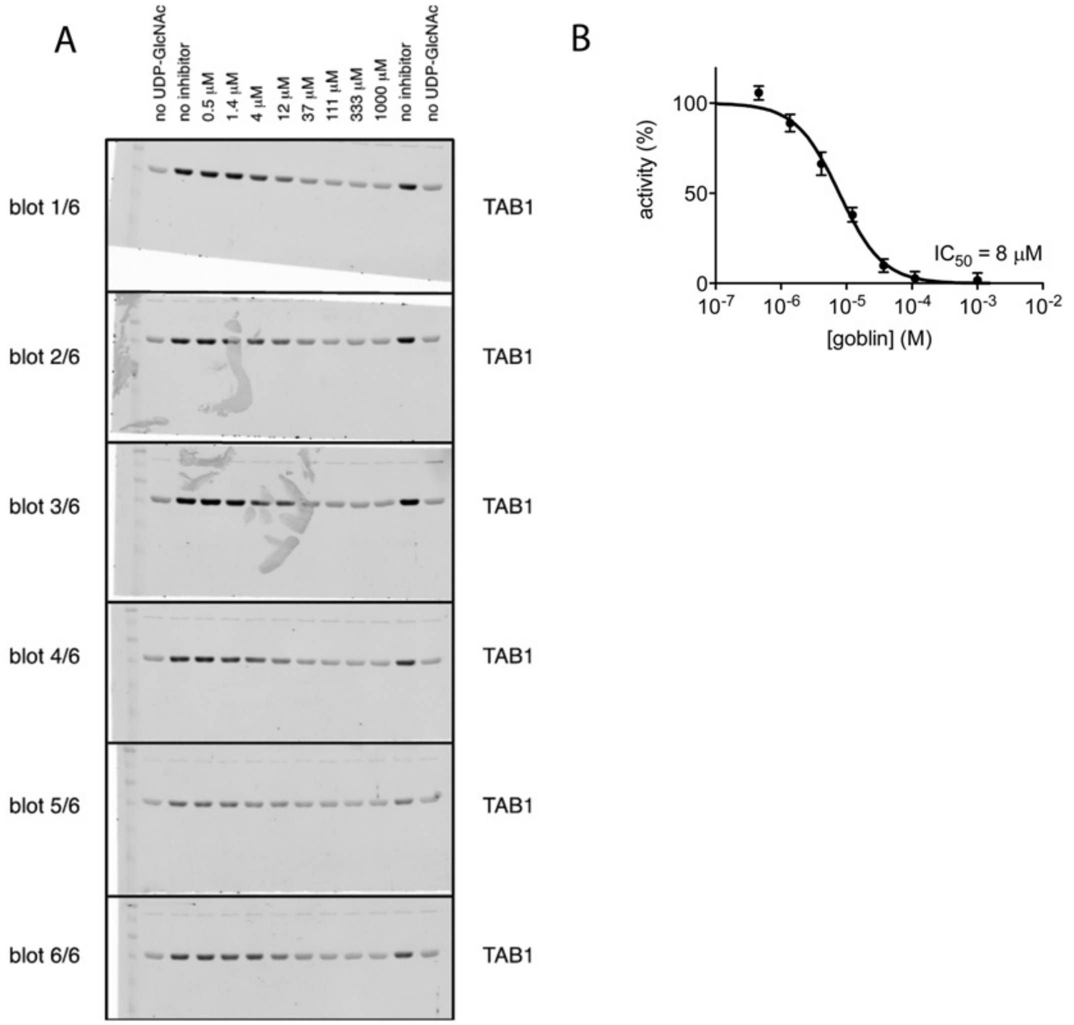

Figure S2 hOGT inhibition by goblin1

In vitro O-GlcNAcylation of TAB1-(7–402) protein was performed in the presence of 10 μM UDP-GlcNAc and 1 mM–0.46 μM goblin1. O-GlcNAc was detected by immunoblotting with a TAB1 O-GlcNAc Ser^{395} site-specific antibody (**A**) [2]. The experiment was performed in six replicates and quantified by densitometric analysis, normalized and fitted to a four-parameter equation for dose-dependent inhibition in GraphPad Prism (http://www.graphpad.com) (**B**). Results are means $\pm$ S.E.M.

156.1, 143.9, 143.8, 141.3, 131.5, 127.7, 127.1, 125.2, 125.1, 120, 119, 71, 69.9, 67.2, 66.3, 60.8, 54.5, 47.1, 32.0. HRMS (*m/z*), $[M+H]^+$ calculated for $C_{24}H_{28}NO_6$, 426.1917; found 4261922.

Compound 5b

$[\alpha]_D = +12.4°$ (*c* 1.00 in chloroform); ^{1}H NMR (500 MHz, [^{2}H]chloroform) δ 7.79 (d, *J* 7.5 Hz, 2H), 7.65 (t, *J* 7.1 Hz, 2H), 7.43 (t, *J* 7.5 Hz, 2H), 7.35 (t, *J* 7.4 Hz, 2H), 5.94 (ddt, *J* 16.5, 10.9, 5.7 Hz, 1H), 5.78 (d, *J* 8.7 Hz, 1H), 5.37 (d, *J* 17.1 Hz, 1H), 5.28 (d, *J* 10.4 Hz, 1H), 4.81–4.65 (m, 2H), 4.62–4.53 (m, 1H), 4.45 (dd, 1H), 4.40 (dd, *J* 10.7, 7.2 Hz, 1H), 4.28 (t, *J* 7.2 Hz, 1H), 3.93 (dd, *J* 9.8, 3.3 Hz, 1H), 3.73 (dd, *J* 9.6, 3.2 Hz, 1H), 3.66 (t, *J* 6.0 Hz, 2H), 3.58–3.46 (m, 2H), 1.78 (s, 2H), 1.66 (dq, *J* 18.0, 6.7 Hz, 4H); ^{13}C NMR (126 MHz, [^{2}H]chloroform) δ 170.1, 156.1, 143.9, 143.8, 141.3, 131.6, 127.7, 127.1, 125.2, 125.1, 120, 118.7, 71.5, 70.6, 67.2, 62.5, 54.6, 47.2, 29.6, 26. HRMS (*m/z*), $[M+H]^+$ calculated for $C_{25}H_{31}NO_6$, 441.2151; found 441.2160.

Compound 6

The reaction was carried out in a 50 ml Falcon centrifuge tube. A solution of di-isopropylamine (3.5 ml, 25 mmol) in hexane (10 ml) was added dropwise to a stirred solution of phosphorus trichloride (1.1 ml, 12.6 mmol) in hexane (30 ml) at -50°C. The reaction was removed from the cooling bath, warmed to room temperature, and further vigorously stirred for 1 h and centrifuged at 3700 ***g*** for 15 min at 4 °C. Approximately 30 ml of the supernatant was transferred into a recovery flask; the residue was resuspended in methyl t-butyl ether (15 ml) and centrifuged as before. The supernatant was added to the recovery flask. The solution was concentrated to give 1.93 g (9.55 mmol, 76 %) of the crude target product (δ 169.9 p.p.m.) as a slightly yellowish liquid. A solution of the above residue (0.97 g, 4.8 mmol) in THF (5 ml) was added to a solution of *p*-methylbenzyl alcohol 1.17 g, 9.6 mmol) and di-isopropylethylamine (2.5 ml, 14.4 mmol) in THF (20 ml) at 0 °C (ice-bath) (reaction was carried out in a 50 ml Falcon centrifuge tube). The reaction was removed from the cooling bath and stirred further for 2 h at room temperature;

© 2014 The Author(s)
The author(s) has paid for this article to be freely available under the terms of the Creative Commons Attribution Licence (CC-BY) (http://creativecommons.org/licenses/by/3.0/) which permits unrestricted use, distribution and reproduction in any medium, provided the original work is properly cited.

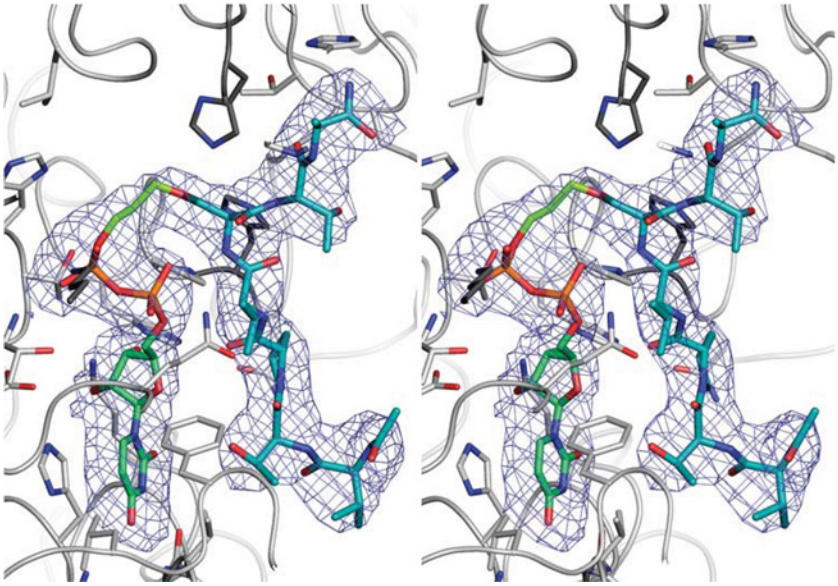

Figure S3 Stereo image of hOGT with bisubstrate inhibitor showing unbiased $F_o - F_c$ electron density after 4-fold averaging (3.5σ)

the precipitate started to fall in 10–15 min. The reaction was diluted with methyl t-butyl ether (total volume 40 ml) and centrifuged as before. The supernatant was collected; the residue was suspended in methyl t-butyl ether (20 ml) and centrifuged again. The supernatants were pooled and concentrated. The residue was partitioned between DCM and 1 M phosphate (pH 7) buffer (pH of the aqueous phase 7 after shaking) and the layers were separated. The organic layer was washed with brine. The aqueous layers were successively extracted with the same portion of DCM. The organic layers were dried, concentrated and dried under vacuum overnight to give 1.7 g of the crude product as a yellowish freely running oil, which was shown to be sufficiently pure by NMR (δ 146 p.p.m.).

Compound 7a

To a solution of **5a** (0.85 g, 2 mmol) and dimethylbenzyl *N*,*N*-di-isopropylphosphoramidite **6** (0.97 g, 2.6 mmol) in acetonitrile (10 ml), 4,5-dicyanoimidazole (0.307 g, 2.6 mmol) was added at room temperature and reaction was stirred for 1 h. The reaction mixture was cooled down to 0 °C (ice-bath) and 3-chloroperbenzoic acid (0.64 g, 2.6 mmol) was added in one portion. The reaction was stirred further for 1 h and quenched by the addition of a 20 % solution of $Na_2S_2O_5$, stirred for 30 min, removed from the cooling bath and diluted with DCM. The layers were separated; the organic layer was washed successively with water and a mixture of concentrated $NaHCO_3$ solution and brine. The aqueous layers were back-extracted with DCM. The combined organic layer was dried and concentrated. The residue was adsorbed on to silica gel and purified by flash chromatography in (petroleum ether/DCM, 4:1, v/v)/ethyl acetate 10–40 % to give 1.21 g (1.7 mmol, 85 %) of the target product as a clear oil.

$[\alpha]_D = +8.3°$(c 1.00 in chloroform);^{1}H NMR (500 MHz, [^{2}H]chloroform) δ 7.79 (d, *J* 7.5 Hz, 2H), 7.70 (dd, *J* 11.5, 7.5 Hz, 2H), 7.45–7.39 (m, 3H), 7.34 (td, *J* 7.5, 1.0 Hz, 2H), 7.29–7.24 (m, 5H), 7.18 (d, *J* 7.6 Hz, 5H), 6.18 (d, *J* 8.6 Hz, 1H), 5.98–5.87 (m, 1H), 5.36 (dq, *J* 17.3, 1.6 Hz, 1H), 5.27 (dq, *J* 10.5, 1.3 Hz, 1H), 5.09–4.97 (m, 5H), 4.75–4.65 (m, 2H), 4.57 (dt, *J* 8.5, 3.3 Hz, 1H), 4.47 (dd, *J* 10.5, 7.2 Hz, 1H), 4.38 (dd, *J* 10.6, 7.4 Hz, 1H), 4.27 (t, *J* 7.3 Hz, 1H), 4.21–4.12 (m, 1H), 4.10 (m,1H), 3.93 (dd, *J* 9.5, 3.5 Hz, 1H), 3.70 (dd, *J* 9.5, 3.1 Hz, 1H), 3.52 (ddt, *J* 26.3, 9.5, 6.1 Hz, 2H), 2.37 (s, 6H), 1.88 (p, *J* 6.0 Hz, 2H); ^{13}C NMR (126 MHz, [^{2}H]chloroform) δ 170, 156.3, 144, 143.9, 141.3, 138.4, 132.93 (d, $J_{C,P}$ 6.4 Hz), 131.7, 129.3, 128.2, 128.2, 127.7, 127, 125.4, 125.3, 112, 118.6, 70.66, 69.27 (dd, $J_{C,P}$ 4.5 Hz), 67.2, 67, 66.1, 64.4 (d, $J_{C,P}$ 5.6 Hz), 54.7, 47.2, 30.24 (d, $J_{C,P}$ 6.1 Hz), 21.3; ^{31}P NMR (202 MHz, [^{2}H]chloroform) δ −0.55. HRMS (*m*/*z*), $[M+H]^+$ calculated for $C_{40}H_{45}NO_9P$ 714.2832; found 714.2826.

Compound 7b

$[\alpha]_D = +5.2°$ (c 2.5 in chloroform); ^{1}H NMR (500 MHz, [^{2}H]chloroform) δ 7.79 (d, *J* 7.5 Hz, 2H), 7.70 (dd, *J* 11.5, 7.5 Hz, 2H), 7.45–7.39 (m, 3H), 7.34 (td, *J* 7.5, 1.0 Hz, 2H), 7.29–7.24 (m, 5H), 7.18 (d, *J* 7.6 Hz, 5H), 6.18 (d, *J* 8.6 Hz, 1H), 5.98–5.87 (m, 1H), 5.36 (dq, *J* 17.3, 1.6 Hz, 1H), 5.27 (dq, *J* 10.5, 1.3 Hz, 1H), 5.09–4.97 (m, 5H), 4.75–4.65 (m, 2H), 4.57 (dt, *J* 8.5, 3.3 Hz, 1H), 4.47 (dd, *J* 10.5, 7.2 Hz, 1H), 4.38 (dd, *J* 10.6, 7.4 Hz, 1H), 4.27 (t, *J* 7.3 Hz, 1H), 4.21–4.12 (m, 1H), 4.07 (q, 2H), 3.9 (dd, *J* 9.3, 3 Hz, 1H), 3.70 (dd, *J* 9.3, 3 Hz, 1H), 3.45 (m, 2H), 2.37 (s, 6H), 1.68 (m, 2H), 1.63 (m, 2H); ^{13}C NMR (126 MHz, [^{2}H]chloroform) δ 170, 156.3, 144, 143.9, 141.3, 138.4, 132.93 (d, $J_{C,P}$ 6.4 Hz), 131.7, 129.3, 128.2, 128.2, 127.7, 127, 125.4, 125.3, 112, 118.6, 70.66, 69.27 (dd, $J_{C,P}$ 4.5 Hz), 67.2, 67, 66.1, 64.4 (d, $J_{C,P}$ 5.6 Hz), 54.7, 47.2, 30.24 (d, $J_{C,P}$ 6.1 Hz), 21.3; ^{31}P NMR (202 MHz, [^{2}H]chloroform) δ −0.9. HRMS (*m*/*z*), $[M+H]^+$ calculated for $C_{41}H_{48}NO_9P$ 729.3067; found 729.306.

Compound 8a

To a solution of **7a** (0.22 g, 0.297 mmol) in THF (3 ml) morpholine (0.05 ml, 0.53 mmol) and $Pd(PPh_3)_4$ [tetrakis (triphenylphosphine)palladium(0)] (0.009 g, 0.0078 mmol) were added sequentially. The reaction was stirred for 1.5 h, quenched by addition of a suspension of Dowex 50WX8-100 H^+ resin in methanol and stirred for 30 min. The resin was filtered off, and the filtrate was concentrated to give 0.195 g (0.29 mmol, 93 %) of the target product as a clear syrup.

© 2014 The Author(s)
The author(s) has paid for this article to be freely available under the terms of the Creative Commons Attribution Licence (CC-BY) (http://creativecommons.org/licenses/by/3.0/) which permits unrestricted use, distribution and reproduction in any medium, provided the original work is properly cited.

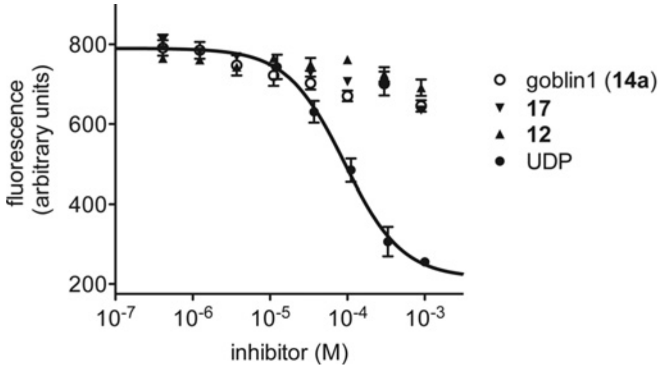

Figure S4 The bacterial GlcNAc transferase NodC is not inhibited by goblin1

*Sm*NodC, a GlcNAc transferase from *Sinorhizobium meliloti* that utilizes UDP-GlcNAc to synthesize chito-oligosaccharides, was assayed to investigate the selectivity of goblin1. A coupled fluorigenic *in vitro* assay was performed in the presence of 1 mM–0.46 μM of goblin1, **12**, **17** and UDP. The assay (details available from D. M. F. v. A. on request) relies on the elongation of 4-methylumbelliferyl-*N*-acetyl-β1,4-glucosamine by the GlcNAc transferase *Sm*NodC, and subsequent hydrolysis of the reaction products by *Aspergillus fumigatus* chitinase B; the release of 4-methylumbelliferone is detected fluorimetrically. Experiments were performed in triplicate, with results being means $\pm$ S.E.M. Dose–response curves were obtained using the 4-parameter IC_{50} equation in the GraphPad Prism program (http://www.graphpad.com). NodC exhibits product inhibition by UDP (IC_{50} 90 μM), but is not inhibited by either the goblin1 inhibitor, or by the goblin1 fragments containing either UDP and linker (**17**), or peptide and linker (**12**).

Table S1 Crystallographic data collection and structure refinement statistics

Values for highest-resolution shell are shown in parentheses.

	hOGT + goblin1
Data collection	
Beamline, wavelength (Å)	ID 23-1, 0.981
Space group	*P*321
Cell dimensions	
a = *b*, *c* (Å)	273.7, 142.6
Resolution (Å)	30–3.15 (3.32–3.15)
R_{merge}	0.136 (0.505)
$I/\sigma I$	9.2 (3.1)
Completeness (%)	99.2 (99.7)
Redundancy	4.3 (4.5)
Refinement	
Resolution (Å)	30–3.15
Number of reflections	452 654
Number of unique reflections	104 870
R_{work}/R_{free}	0.197/0.213
Number of atoms	
Protein	22 056
Ligand	312
B-factors	
Protein	54.5
Ligand	49.8
RMSDs	
Bond lengths (Å)	0.011
Bond angles (°)	1.34

$[\alpha]_D = +24.3°$ (*c* 1.00 in chloroform); ^{1}H NMR (500 MHz, [^{2}H]chloroform) δ 7.79–7.75 (m, 2H), 7.65 (dd, *J* 11.0, 7.5 Hz, 2H), 7.41 (td, *J* 7.4, 1.0 Hz, 2H), 7.33 (tt, *J* 7.4, 1.0 Hz, 2H), 7.27–7.20 (m, 4H), 7.16 (t, *J* 8.2 Hz, 4H), 6.31 (d, *J* 8.2 Hz, 1H), 5.11–4.95 (m, 4H), 4.61 (dt, *J* 8.2, 3.0 Hz, 1H), 4.41 (dd, *J* 10.5, 7.2 Hz, 1H), 4.31 (dd, *J* 10.5, 7.5 Hz, 1H), 4.24–4.14 (m, 2H), 4.10 (dq, *J* 11.2, 5.9 Hz, 1H), 3.92 (dd, *J* 9.5, 3.3 Hz, 1H), 3.79 (dd, *J* 9.5, 2.9 Hz, 1H), 3.58 (dt, *J* 10.4, 5.4 Hz, 1H), 3.47 (dt, *J* 9.8, 6.0 Hz, 1H), 2.37 (s, 3H), 2.35 (s, 3H), 1.87–1.80 (m, 2H); ^{13}C NMR (126 MHz, [^{2}H]chloroform) δ 172.1, 156.2, 144, 143.9, 141.3, 138.5, 132.6, 132.2, 130.1, 129.3, 128.27, 128.2, 127.7, 127.1, 125.35, 125.3, 119.9, 69.7 (d, $J_{C,P}$ 5.5 Hz), 69.6 (d, $J_{C,P}$ 5.5 Hz), 69.6, 67.2, 66.8, 64.8 (d, $J_{C,P}$ 5.1 Hz), 54.6, 47.1, 30.2, 30.1, 21.2; ^{31}P NMR (202 MHz, [^{2}H]chloroform) δ −0.84. HRMS (*m/z*), $[M-H]^-$ calculated for $C_{37}H_{39}NO_9P$, 672.2362; found 672.2357.

Compound 8b

$[\alpha]_D = +19.2°$ (*c* 1.00 in chloroform); ^{1}H NMR (500 MHz, [^{2}H]chloroform) δ 7.79–7.75 (m, 2H), 7.65 (dd, *J* 11.0, 7.5 Hz, 2H), 7.41 (td, *J* 7.4, 1.0 Hz, 2H), 7.33 (tt, *J* 7.4, 1.0 Hz, 2H), 7.27–7.20 (m, 4H), 7.16 (t, *J* 8.2 Hz, 4H), 6.31 (d, *J* 8.2 Hz, 1H), 5.11–4.95 (m, 4H), 4.61 (dt, *J* 8.2, 3.0 Hz, 1H), 4.41 (dd, *J* 10.5, 7.2 Hz, 1H), 4.31 (dd, *J* 10.5, 7.5 Hz, 1H), 4.24–4.14 (m, 2H), 4.10 (dq, *J* 11.2, 5.9 Hz, 1H), 3.92 (dd, *J* 9.5, 3.3 Hz, 1H), 3.79 (dd, *J* 9.5, 2.9 Hz, 1H), 3.58 (dt, *J* 10.4, 5.4 Hz, 1H), 3.47 (dt, *J* 9.8, 6.0 Hz, 1H), 2.37 (s, 3H), 2.35 (s, 3H), 1.8 (m, 1H), 1.7 (m, 1H), 1.60 (m, 2H); ^{13}C NMR (126 MHz, [^{2}H]chloroform) δ 172.1, 156.2, 144, 143.9, 141.3, 138.5, 132.6, 132.2, 130.1, 129.3, 128.27, 128.2, 127.7, 127.1, 125.35, 125.3, 119.9, 69.7 (d, $J_{C,P}$ 5.5 Hz), 69.6 (d, $J_{C,P}$ 5.5 Hz), 69.6, 67.2, 66.8, 64.8 (d, $J_{C,P}$ 5.1 Hz), 54.6, 47.1, 30.2, 30.1, 21.2; ^{31}P NMR (202 MHz, [^{2}H]chloroform) δ −0.94. HRMS (*m/z*), $[M+H]^+$ calculated for $C_{38}H_{44}NO_9P$, 689.2754; found 689.2781.

Compound 9

To a solution of **5a** (0.123 g; 0.29 mmol) in DCM (5 ml) and DIPEA (0.116 ml, 0.7 mmol), TBSCl (t-butyldimethylsilyl chloride) (0.075 g, 0.5 mmol) and DMAP (4-dimethylaminopyridine) (0.04 g, 0.035 mmol) were added sequentially. The reaction mixture was stirred for 16 h at room temperature. The reaction was quenched by the addition of methanol, stirred for 30 min and concentrated. The residue was partitioned between ethyl acetate and 10 % aqueous citric acid solution and the layers were separated. The organic layer was washed successively with water and a mixture of saturated $NaHCO_3$ solution and brine. The aqueous layer was additionally extracted with the same portion of ethyl acetate. The combined organic layer was dried and concentrated. The residue was purified by flash chromatography on silica; gradient elution petroleum ether/ether 10–30 % to give 0.14 g (0.26 mmol, 90 %) of the target product as a clear syrup.

$[\alpha]_D = +7.4°$ (*c* 1.23 in chloroform) ^{1}H NMR (500 MHz, [^{2}H]chloroform) δ 7.79–7.67 (m, 2H), 7.63–7.50 (m, 2H), 7.40–7.28 (m, 2H), 7.34–7.23 (m, 2H), 5.93–5.81 (m, 1H), 5.62 (d, *J* 8.8 Hz, 1H), 5.29 (dq, *J* 17.2, 1.6 Hz, 1H), 5.20 (dq, *J* 10.6, 1.3 Hz, 1H), 4.63 (ddt, *J* 5.7, 4.2, 1.5 Hz, 2H), 4.48 (dt, *J* 8.8, 3.2 Hz, 1H), 4.39 (dd, *J* 10.6, 7.1 Hz, 1H), 4.31 (dd, *J* 10.6, 7.3 Hz, 1H), 4.20 (t, *J* 7.2 Hz, 1H), 3.86 (dd, *J* 9.5, 3.1 Hz, 1H), 3.62 (tdd, *J* 12.8, 9.9, 4.7 Hz, 3H), 3.56–3.43 (m, 2H), 1.71 (p, *J* 6.2 Hz, 2H), 0.85 (s, 9H), 0.00 (s, 6H). ^{13}C NMR (126 MHz, [^{2}H]chloroform) δ 170.12, 156.09, 143.95, 143.77, 141.29, 127.71, 127.08, 125.21, 125.15, 119.99, 118.54, 77.30, 77.26, 77.05, 76.79, 70.57, 68.28, 67.24, 66.10, 59.67, 54.57, 47.13, 32.63, 25.95, 18.34, −5.33. HRMS (*m/z*), $[M+H]^+$ calculated for $C_{30}H_{42}NO_6Si$ 540.2781; found 540.2831.

Compound 10

To a solution of **9** (0.13 g, 0.24 mmol) in THF (2.5 ml) morpholine (0.04 ml, 0.454 mmol) and $Pd(PPh_3)_4$ (0.012 g, 0.024 mmol)

© 2014 The Author(s)
The author(s) has paid for this article to be freely available under the terms of the Creative Commons Attribution Licence (CC-BY) (http://creativecommons.org/licenses/by/3.0/) which permits unrestricted use, distribution and reproduction in any medium, provided the original work is properly cited.

were added sequentially. The reaction was stirred for 1.5 h; TLC (petroleum ether/DCM, 4:1, v/v)/ethyl acetate 40 % and DCM/methanol 10 % showed disappearance of the starting material and formation of a more polar new product. The reaction was quenched by addition of a suspension of Dowex 50WX8-100 H^+ resin in methanol and stirred for 10 min. The resin was filtered off, and the filtrate was concentrated to give 0.123 g (0.24 mmol, quant) of the target product as a yellow syrup.

^{1}H NMR (500 MHz, [^{2}H]chloroform) δ 7.70 (d, *J* 7.6 Hz, 2H), 7.57 (t, *J* 7.2 Hz, 2H), 7.34 (t, *J* 7.5 Hz, 2H), 7.26 (q, *J* 7.4, 6.4 Hz, 3H), 5.71 (d, *J* 8.5 Hz, 1H), 4.58–4.37 (m, 2H), 4.37–4.29 (m, 2H), 4.19 (t, *J* 7.2 Hz, 1H), 3.63 (p, *J* 10.8 Hz, 3H), 3.51 (dt, *J* 10.0, 4.6 Hz, 2H), 1.72 (dd, *J* 8.3, 4.0 Hz, 2H), 0.84 (s, 9H), 0.00 (s, 6H). HRMS (*m/z*), $[M+H]^+$ calculated for $C_{43}H_{71}N_{10}O_{23}P_2$ 500.2468; found 500.2461.

Compound 11a

Peptide synthesis was performed on RinkAmide MBHA (4-methylbenzhydrylamine) low-load resin (0.38 mmol/g, 0.13 g, 0.05 mmol) (Novabiochem). Standard couplings were performed using 5 equiv. of amino acid, 5 equiv. of HCTU [*O*-(1*H*-6-chlorobenzotriazole-1-yl)-1,1,3,3-tetramethyluronium hexafluorophosphate] and 10 equiv. of DIPEA in a microwave-heated reactor (CEM Liberty microwave peptide synthesizer) for 5 min at 70 °C. Deprotections were performed with 20 % piperidine in DMF initially for 0.5 min at 70 °C and then with a fresh portion of deprotection mixture for 3 min at 70 °C. Addition of the 'stretched serine' building block **8a** was performed using 2.5 equiv. of the amino acid for 20 min at 60 °C. After this point, deprotections were performed initially for 5 min at room temperature and then with a fresh portion of deprotection mixture for 15 min at room temperature. The peptide was cleaved from the resin with a mixture of TFA/water/TIPS (tri-isopropylsilane) 92.5:5:2.5, by vol. (2 ml) for 3 h. The cleavage mixture was filtered into the centrifuge tube containing cold (0 °C) diethyl ether (40 ml). The resin was additionally twice washed with 1 ml of TFA for 5 min each and the washings were added to the same tube. The tube was kept in the freezer (−18 °C) for 16 h. The precipitated peptide was centrifuged at 3700 ***g*** at 4 °C for 15 min; the supernatant was discarded. The pellet was suspended in diethyl ether and centrifuged again. The procedure was repeated once more. The pellet was blow-dried with argon, dissolved in 20 % aqueous acetic acid and freeze-dried to give 0.025 g (0.03 mmol) of the crude peptide. HRMS (*m/z*), $[M-H]^-$ calculated for $C_{34}H_{60}N_8O_{15}P$, 851.3916; found 851.3907.

Compound 11b

HRMS (*m/z*), $[M-H]^-$ calculated for $C_{35}H_{62}N_8O_{15}P$, 865.4072; found 865.4100

Compound 12

The peptide was synthesized using 'stretched serine' building block **10** (2.5 equiv.) according to the general procedure.

^{1}H NMR (500 MHz, ^2H_2O) δ 4.61–4.56 (m, 2H), 4.45–4.41 (m, 1H), 4.31 (d, *J* 4.7 Hz, 1H), 4.30–4.25 (m, 1H), 4.19 (tt, *J* 6.5, 5.4 Hz, 1H), 4.13–4.03 (m, 3H), 3.91–3.83 (m, 1H), 3.76 (d, *J* 5.6 Hz, 2H), 3.69 (ddd, *J* 10.7, 8.6, 6.8 Hz, 1H), 3.58 (dtd, *J* 10.1, 8.5, 7.5, 2.6 Hz, 4H), 2.31–2.21 (m, 1H), 2.09–2.02 (m, 2H), 2.01 (s, 2H), 1.95 (dt, *J* 13.1, 7.3 Hz, 1H), 1.87 (dq, *J* 13.7, 6.9 Hz, 1H), 1.76 (td, *J* 6.4, 1.4 Hz, 2H), 1.37 (d, *J* 7.2 Hz, 3H), 1.22 (d, *J* 6.4 Hz, 3H), 1.17 (d, *J* 6.4 Hz, 3H), 0.94 (d, *J* 6.7 Hz, 3H), 0.93 (d, *J* 6.8 Hz, 3H), 0.90 (d, *J* 6.5 Hz, 3H), 0.88 (d, *J* 6.8 Hz, 3H).

^{13}C NMR (126 MHz, ^2H_2O) δ 177.46, 174.38, 174.09, 173.92, 173.64, 171.69, 171.21, 170.02, 69.12, 68.22, 67.05, 67.00, 60.37, 59.59, 59.49, 58.87, 58.69, 57.06, 53.59, 49.50, 48.50, 31.22, 30.16, 30.05, 29.50, 24.69, 21.65, 18.74, 18.66, 18.45, 18.38, 17.80, 17.60, 16.74. HRMS MS–TOF (positive): *m/z* 773.4434, calculated for $C_{34}H_{61}N_8O_{12}$ 773.4409, $[M+H]^+$

Compound 14a

2′,3′-*O*-diacetyl-UMP triethylammonium salt **13** (0.17 g, 0.3 mmol) was rendered dry by co-evaporation with DMF (3 ml), dissolved in DMF (3 ml) and treated with 1,1′-carbonylbisimidazole (0.096 g, 0.59 mmol). The reaction was kept for 16 h at room temperature, quenched by addition of methanol (0.032 ml, 0.77 mmol), kept for 30 min at room temperature and concentrated. ^{31}P NMR showed the presence of a single principal product [^{31}P NMR (202 MHz, ^2H_2O) δ −10.4]; assumed to be phosphoimidazolide. A solution of the crude peptide **11a** (0.025 g, 0.03 mmol) in DMF (2 ml) was treated with triethylamine (0.03 ml) and concentrated. To the residue was added a solution of the crude phosphoimidazolide in DMF (2 ml) and the reaction was kept for 20 h at room temperature. The reaction products were concentrated, dissolved in a mixture of methanol (2 ml), water (0.5 ml) and triethylamine (0.25 ml), and kept for 16 h at room temperature. The reaction was concentrated, dissolved in acetonitrile/water 95:5 (v/v) in 0.1 % TFA (10 ml), filtered through a 0.45 μm syringe filter and purified by HPLC (Waters 19 × 100 Peptide separation technology column, gradient 5–95 % acetonitrile in 0.1 % TFA, detection at 214/254 nm). The fractions containing product were pooled and freeze-dried. The powder was dissolved in 0.25 M NH_4HCO_3 buffer and freeze-dried to give 0.015 g (0.013 mmol) of the target product as a fluffy powder.

^{1}H NMR (500 MHz, ^2H_2O) δ 7.89 (d, *J* 8.1 Hz, 1H), 5.94–5.91 (m, 1H), 5.90 (d, *J* 8.1 Hz, 1H), 4.54 (td, *J* 6.0, 5.6, 2.4 Hz, 2H), 4.41 (dd, *J* 8.3, 6.1 Hz, 1H), 4.32–4.28 (m, 2H), 4.28–4.23 (m, 2H), 4.22 (d, *J* 2.8 Hz, 1H), 4.19–4.16 (m, 1H), 4.16–4.13 (m, 1H), 3.95 (q, *J* 6.6 Hz, 2H), 3.87–3.81 (m, 1H), 3.74 (d, *J* 5.5 Hz, 2H), 3.66 (dt, *J* 10.2, 7.2 Hz, 1H), 3.58 (q, *J* 6.3 Hz, 2H), 2.30–2.20 (m, 1H), 2.07–1.89 (m, 2H), 1.98 (s, 2H), 1.85 (q, *J* 7.3, 6.3 Hz, 3H), 1.34 (d, *J* 7.2 Hz, 3H), 1.19 (d, *J* 6.3 Hz, 2H), 1.15 (d, *J* 6.5 Hz, 3H), 0.91 (dd, 6H), 0.87 (dd, 6H); ^{13}C NMR (126 MHz, ^2H_2O) δ 177.5, 174.4, 174.1, 173.9, 173.7, 171.8, 171.3, 170, 166.1, 151.8, 141.7, 102.6, 88.3, 83.2 (d, $J_{C,P}$ 8.9 Hz), 69.7, 69, 67.9, 67, 65 (d, $J_{C,P}$ 5.4 Hz), 63.6 (d, $J_{C,P}$ 6.2 Hz), 60.3, 59.6, 59.4, 59, 57, 53.8, 53.7, 49.5, 48.5, 30.1, 30, 29.8 (d, $J_{C,P}$ 7.1 Hz), 29.5, 24.7, 21.6, 18.72, 18.7, 18.44, 18.4, 17.7, 17.6, 16.7; ^{31}P NMR (202 MHz, ^2H_2O) δ −11.08 (d, $J_{P\alpha,P\beta}$ 26.6 Hz), −11.56 (d, $J_{P\alpha,P\beta}$ 26.6 Hz). HRMS (*m/z*), $[M-H]^-$ calculated for $C_{43}H_{71}N_{10}O_{23}P_2$ 1157.4169; found 1157.4171.

Compound 14b

^{1}H NMR (500 MHz, ^2H_2O) δ 7.91 (d, *J* 8.1 Hz, 1H), 5.93 (d, *J* 4.6 Hz, 1H), 5.91 (d, *J* 8.4 Hz, 1H), 4.58–4.52 (m, 1H), 4.41 (dd, *J* 8.2, 6.1 Hz, 1H), 4.33–4.29 (m, 2H), 4.28 (d, *J* 4.5 Hz, 1H), 4.26 (d, *J* 7.3 Hz, 1H), 4.24–4.20 (m, 1H), 4.20–4.15 (m, 1H), 4.13 (dd, *J* 5.6, 3.0 Hz, 1H), 4.09–4.03 (m, 1H), 4.08 (dd, *J* 9.7, 7.4 Hz, 2H), 3.86 (dq, *J* 15.9, 6.8, 6.0 Hz, 3H), 3.73 (d, *J* 5.6 Hz, 2H), 3.67 (dt, *J* 10.2, 7.1 Hz, 1H), 3.54–3.46 (m, 2H), 2.29–2.21 (m, 1H), 2.08–2.00 (m, 2H), 1.99 (s, 3H), 1.97–1.90 (m, 1H), 1.82–1.89 (m, 1H), 1.57 (m, 4H), 1.35 (d, *J* 7.2 Hz, 3H), 1.20 (d,

© 2014 The Author(s)
The author(s) has paid for this article to be freely available under the terms of the Creative Commons Attribution Licence (CC-BY) (http://creativecommons.org/licenses/by/3.0/) which permits unrestricted use, distribution and reproduction in any medium, provided the original work is properly cited.

J 6.4 Hz, 3H), 1.15 (d, *J* 6.4 Hz, 3H), 0.91 (dd, 6H), 0.87 (dd, 6H); ^{13}C NMR (126 MHz, ^2H_2O) δ 177.5, 174.4, 174.1, 173.9, 173.6, 171.7, 171.2, 167, 166.1, 151.8, 141.7, 102.7, 88.4, 83.3 (d, *J* 9.0 Hz), 73.8, 71, 69.7, 68.9, 67 (d, $J_{C,P}$ 7.2 Hz), 66.3 (d, $J_{C,P}$ 5.6 Hz), 64.9 (d, $J_{C,P}$ 5.0 Hz), 60.3, 59.6, 59.5, 59, 57, 53.6, 49.5, 48.5, 30.1, 30, 29.5, 26.4 (d, $J_{C,P}$ 7.2 Hz), 25.1, 24.7, 21.6, 18.73, 18.66, 18.43, 18.4, 17.8, 17.6, 16.7; ^{31}P NMR (202 MHz, ^2H_2O) δ -10.97 (d, $J_{P\alpha,P\beta}$ 27.2 Hz), −11.53 (d, $J_{P\alpha,P\beta}$ 27.2 Hz). HRMS (*m/z*), $[M-H]^-$ calculated for $C_{44}H_{73}N_{10}O_{23}P_2$ 1171.4325; found 1171.4343.

Compound 17

To a cold (0°C) solution of 3-methoxypropanol **15** (0.018 g, 0.25 mmol) in acetonitrile (5 ml) and PMBPA [di-(*p*-methoxybenzyl)-*N,N*-diisopropylphosphoramidite] **16** [1] (0.26 g, 0.5 mmol) was added DCI (0.059 g, 0.5 mmol). After 30 min, a 0.5 M solution of iodine in pyridine (1.1 ml, 0.55 mmol) was added to the reaction mixture, to produce a tan-coloured solution, which was stirred further for 1 h. Then, 2′,3′-*O*-diacetyl-UMP triethylammonium salt **13** (0.14 g, 0.25 mmol) in 2.5 ml of acetonitrile was added and stirred for 16 h at room temperature. The reaction was concentrated and dissolved in a 5:2:1 (by vol.) methanol/H_2O/triethylamine mixture (8 ml) and stirred for 16 h at room temperature. The reaction mixture was concentrated, diluted with water and extracted with chloroform. The mixture was centrifuged at 3700 ***g*** for 15 min at 4°C. The clear layers were separated; the organic layer was extracted with water and centrifuged once more. The combined aqueous layer was extracted with chloroform, centrifuged and concentrated with the addition of n-butanol to give an oily residue. This was purified by size-exclusion chromatography (Bio-Gel P2 fine; column 2.6 cm × 100 cm; flow rate 0.4 ml/min; elution with 0.25 M NH_4HCO_3). The fractions containing the product were pooled and freeze-dried to give 0.05 g (0.1 mmol, 40 %) of the target product as a white fluffy solid.

^{1}H NMR (500 MHz, ^2H_2O) δ 7.76 (d, *J* 8.1, 1H, H-6), 5.86 (d, 1H, H-1′), 5.80 (d, *J* 8.1, 1H, H-5), 4.28–4.18 (m, 2H, H-2′, H-3′), 4.15–4.08 (m, 2H, H-4′, H-5a′), 4.05 (ddd, *J* 11.6, 5.4, 2.8 Hz, 1H, H-5b′), 3.86 [q, *J* 6.6 Hz, 2H, $CH_3OCH_2CH_2C\underline{H_2}OP(O)$], 3.42 [t, *J* 6.5 Hz, 1H, 2H, $CH_3OC\underline{H_2}CH_2CH_2OP(O)$], 3.21 (s, 3H, $C\underline{H_3}O$), 1.77 [dq, *J* 6.4 Hz, 2H, $CH_3OCH_2C\underline{H_2}CH_2OP(O)$]. ^{13}C NMR (126 MHz, ^2H_2O) δ 170.37, 154.80, 141.15 (C-6), 102.77 (C-5), 88.56 (C-1′), 82.82 (d, $J_{C,P}$ 9 Hz, C-4′), 73.66 (C-2′/C-3), 69.55 (C-2′/C-3), 68.93 [$CH_3O\underline{C}H_2CH_2CH_2OP(O)$], 64.90 (d, $J_{C,P}$ 5.5 Hz, C-5′), 63.50 [d, $J_{C,P}$ 5.5 Hz, $CH_3OCH_2CH_2\underline{C}H_2OP(O)$], 57.75 ($\underline{C}H_3O$), 29.58 [d, $J_{C,P}$ 7.4 Hz, $CH_3OCH_2\underline{C}H_2CH_2OP(O)$]. ^{31}P NMR (202 MHz, ^2H_2O) δ − 11.14 (d, *J* 19.7 Hz), − 11.66 (d, *J* 19.8 Hz). HRMS (*m/z*), $[M-H]^-$ calculated for $C_{13}H_{21}N_2O_{13}P_2$ 475.0519; found 475.0531.

Received 25 September 2013/13 November 2013; accepted 21 November 2013
Published as BJ Immediate Publication 21 November 2013, doi:10.1042/BJ20131272

REFERENCES

1 van der Heden van Noort, G. J., Verhagen, C. P., van der Horst, M. G., Overkleeft, H. S., van der Marel, G. A. and Filippov, D. V. (2008) A versatile one-pot procedure to phosphate monoesters and pyrophosphates using di(p-methoxybenzyl)-N,N-diisopropylphosphoramidite. Org. Lett. **10**, 4461–4464
2 Pathak, S., Borodkin, V. S., Albarbarawi, O., Campbell, D. G., Ibrahim, A. and van Aalten, D. M. (2012) O-GlcNAcylation of TAB1 modulates TAK1-mediated cytokine release. EMBO J. **31**, 1394–1404

© 2014 The Author(s)
The author(s) has paid for this article to be freely available under the terms of the Creative Commons Attribution Licence (CC-BY) (http://creativecommons.org/licenses/by/3.0/) which permits unrestricted use, distribution and reproduction in any medium, provided the original work is properly cited.
